# Non-tuberculous mycobacteria lung disease due to *Mycobacterium chimaera* in a 67-year-old man treated with immune checkpoint inhibitors for lung adenocarcinoma: infection due to dysregulated immunity?

**DOI:** 10.1186/s12879-023-08537-w

**Published:** 2023-09-04

**Authors:** Cecilia Azzarà, Andrea Lombardi, Andrea Gramegna, Margherita Ori, Andrea Gori, Francesco Blasi, Alessandra Bandera

**Affiliations:** 1 Infectious Diseases Unit, Foundation IRCCS Ca’ Granda Ospedale Maggiore Policlinico, Milan, Italy; 2 Department of Pathophysiology and Transplantation, University of Milan, Via Francesco Sforza 35, Milan, Italy; 3 Internal Medicine Department, Respiratory Unit and Cystic Fibrosis Adult Center, Foundation IRCCS Ca’ Granda Ospedale Maggiore Policlinico, Milan, Italy

**Keywords:** Immune checkpoint inhibitors, Non-tuberculous mycobacteria, Immune-related adverse events, *Mycobacterium chimaera*, NTM-LD

## Abstract

Immune checkpoint inhibitors (ICIs) are drugs growingly employed in cancer immunotherapy which have significantly improved the prognosis of several tumours. ICIs act by restoring the “exhausted” immune system and increasing the number of T cells active against pathogens losing tolerogenic signalling, which has been linked to an increased risk of infectious events. We present the case of a 67-year-old man with locally advanced lung adenocarcinoma treated with the anti-PD-L1 durvalumab. Three months after immunotherapy started, an apparent radiological progression was found with elements suggesting a parenchymal superinfection associated with weight loss, asthenia, and sputum emission. A bronchoalveolar lavage resulted positive for *Mycobacterium chimaera*, and treatment with amikacin iv (for eight weeks) and daily azithromycin, ethambutol, and rifampicin was started. Thirteen months after treatment started, the patient is alive with a stable lung condition. The case highlights the risk of non-tuberculous mycobacteria lung disease (NTM-LD) in patients receiving ICIs treatment. We hypothesise that durvalumab induced an exaggerated immune response toward the mycobacteria, leading to immunopathology and overt clinical manifestations. Clinicians should be aware of this possibility in patients receiving ICIs developing new signs/symptoms related to the respiratory tract, especially in countries with a high prevalence of NTM-LD.

## Introduction

Immune checkpoint inhibitors (ICIs) are growingly employed in cancer therapy. ICIs bind to cytotoxic T-lymphocyte-associated protein 4 (CTLA-4), programmed cell death protein 1 (PD-1) or programmed death ligand 1 (PD-L1), overriding the inhibitory signal generated by these proteins, and re-established a “depleted’’ immune response against neoplastic antigens. A similar condition of immune exhaustion has been described for some chronic infections, including NTM infections [1, 2].

ICI therapy has long been considered a risk factor for developing infections in patients receiving it. Firstly, ICI therapy sometimes requires using glucocorticoids or other immunosuppressive drugs to manage immune-related adverse events (IRAE), which can lead to opportunistic infections (such as reactivation of *P. jirovecii* or CMV). However, another mechanism has been hypothesised that attributes to ICI’s role in a new pattern of infections resulting from their effect in causing unregulated hyperinflammatory immunity [3]. The activity of the active immune system (where T cells are inhibited in their hyperinflammatory activity by PD-1/PD-L1 activity) plays a crucial role in controlling chronic respiratory infections, including tuberculosis, by maintaining a balance between the pathogen and the host [4]. Conversely, when immunomodulating therapy with ICI is initiated in oncology patients to target neoplastic cells, it disrupts the homeostasis of chronic infections (due to the upregulation of T cells in the absence of physiological inhibition), leading to clinically relevant manifestations [3].

The impact of ICI therapy on the risk of developing non-tuberculous mycobacterial (NTM) infections is still unclear. The presence of a dysfunctional immune response to NTM, showing the characteristics of immune exhaustion, has been clearly described, thus providing the theoretical explanation for the occurrence of immunopathology among ICI-treated patients during NTM infection [5]. The cases of NTM infections occurring during ICIs therapy and involving the lungs are mainly caused by NTM belonging to *Mycobacterium avium* complex (MAC) [6]. Recent studies analysed the probability of NTM-LD stratified by subspecies of MAC, ascertaining the possible causal role of *M. chimaera*, even if it appears less virulent than *M. intracellulare* and *M. avium* [7]. A large study performed in the US has associated the development of NTM-LD due to *M. chimaera* with immunosuppression [8]. Interestingly, in the same study, malignancy was more prevalent among patients with *M. chimaera* infection, even if that does not reach statistical significance [8]. Interestingly, Fujita et al. described a possible association between ICI therapy and the development of NTM-LD in three Japanese patients in 2020 [9]. A retrospective review of the US Food and Drug Administration Adverse Events Reporting System (FAERS) identified 13 cases of NTM infection following treatment with PD-(L)1 inhibitors. The reporting odds ratio (ROR) of infection between PD-(L)1 inhibitors and other drugs was 5.49 (95% CI: 3.15–9.55, p < 0.0001), showing an increased risk of NTM infection associated with ICI treatment [10].

Considering the increasing number of patients receiving ICI and the rising global prevalence of NTM-LD, an increase in NTM-LD incidence during ICIs treatment is expected. Here we report the first case of NTM-LD due to *Mycobacterium chimaera* in a patient receiving cancer immunotherapy with durvalumab.

## Case report

**Fig. 1:**
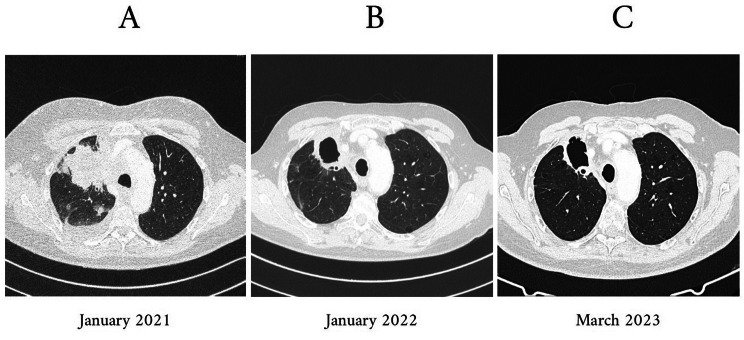
Chest CT images performed (**A**) during the patient’s diagnosis of cancer in January 2021, (**B**) 1 year later, in January 2022, before starting NTM-LD treatment and approximately three months after discontinuation of durvalumab therapy, and (**C**) in March 2023, thirteenth month after the start of NTM-LD treatment and five months off immunomodulatory therapy with ICI.

A 67-year-old man, former computer engineer - BMI 22 and active smoker, was diagnosed with locally advanced right upper lobe lung adenocarcinoma after a radiologic investigation was performed for the appearance of a mediastinal syndrome. Lung cancer was diagnosed in January 2021; a computed tomography (CT) (Fig. 1–A) showed in the right upper lobe, there was a coarse solid formation, exhibiting heterogeneous contrast enhancement and a large central necrotic portion, measuring approximately 6 × 9 cm in axial dimensions. It had spiculated margins and showed invasive growth beyond the lung, infiltrating the mediastinal pleura with the involvement of the middle mediastinum. Only tumour cells for lung adenocarcinoma were found at the first diagnostic transthoracic needle biopsy, with negative microbiological investigations. The patient received four cycles of combined carboplatin and pemetrexed from February 2021 to April 2021, in addition to radiotherapy, starting from the second cycle of chemotherapy. In May 2021, a follow-up CT scan was performed, which showed a recurrence of the oncologic disease, with the additional description of a cavity within the lesion located in the right apex. Due to disease evaluation, immunohistochemistry for PD-L1 was performed, revealing a tumour proportion score of 10%. The patient started in June 2021 second-line systemic immunotherapy with the anti-PD-1 durvalumab alone. CT images acquired during the third month of durvalumab therapy (September 2021) revealed more extensive consolidations in the right upper lobe, still exhibiting cavitated components. These cavities show thicker walls compared to the previous examination, and there is also a more prominent presence of air-fluid levels compared to the previous examination”. Then, ICI treatment was stopped due to suspicion of a concurrent mycobacterial or fungal infection. The subsequent bronchoalveolar lavage, done in the right upper lobe in October 2021, culture was positive only for *Mycobacterium chimaera* (the antimicrobial susceptibility test is reported in **Table **1); microscopic examination for acid-fast bacilli (AFB) and PCR testing for Mycobacterium tuberculosis (MTB) were negative. Bacterial and fungal culture test was negative; galactomannan antigen ad *Aspergillus* PCR was negative; histological examination for malignant cells on BAL was negative. Clinical manifestations were compatible with both mycobacterial infection and lung cancer (weight loss, occasional blood emission in sputum, asthenia, and cough). According to the ATS/ERS/ESCMID/IDSA Clinical Practice Guideline published in 2020, our patient met the diagnostic criteria for the NTM pulmonary disease: pulmonary and systemic symptoms; cavitary characteristic opacities on CT and exclusion of other infective diagnoses; positive culture results from at least one bronchial wash or lavage [11]. In January 2022, the patient underwent a repeat CT scan of the brain, chest, and abdomen to assess any potential progression of the oncological disease after approximately two months of suspension of anti-tumour therapy and to evaluate the infectious origin of the excavated lesion. The radiological investigation (Fig. 1-B) reveals a significant volumetric reduction of the right upper lobe, within which consolidations are observed, some with air bronchograms and increased areas of cavitation. Some of these cavities are in communication with bronchial structures, raising suspicion of superimposed inflammatory/infectious.

**Table 1:** Antimicrobial susceptibility testing of the isolate. MICs interpreted according to the Clinical and Laboratory Standards Institute clinical breakpoints. (MIC, minimum inhibitory concentration)

Antimicrobial	Interpretation	MIC (mg/L)
**Amikacin**	Susceptible	16
**Clarithromycin**	Susceptible	4
**Linezolid**	Resistant	32
**Moxifloxacin**	Resistant	4

Considering all the above, the multidisciplinary team (infectious diseases specialist, pneumologist, and oncologist), with the patient’s opinion, decided to start in February 2022 an antimycobacterial treatment with amikacin administered intravenously for eight weeks and ethambutol (ETB), rifampicin (RIF), and azithromycin (AZT) daily. According to the ATS/ERS/ESCMID/IDSA Clinical Practice Guideline published in 2020, in patients with cavitary MAC pulmonary disease is suggested that parenteral amikacin be included in the initial treatment regimen and administration of at least 2–3 months of it was the best balance between risks and benefits [11]. Therapeutic drug monitoring (TDM) was undertaken to monitor the patient for potential aminoglycoside toxicity. An audiometric examination was performed before starting antibiotic therapy and at four weeks. Ophthalmic evaluation was conducted at baseline.

Under this treatment, the 13-month follow-up CT (Fig. 1- C, March 2023) demonstrated no progression in cavitary lung lesions even if the ICI treatment was restarted in March 2022 and stopped in October 2022. No more isolation of *Mycobacterium* spp. has occurred at sputum cultures performed until January 2023. At month 13 of continuous antimicrobial therapy, the patient was in good health and had no more radiological evolution (stability of the excavation) even though he had stopped oncologic therapy for five months. Figure 1 shows the evolution of the radiologic pattern.

## Discussion

NTM infections occurring during immunotherapy with ICIs appear to be an emerging issue, with several cases identified over the last years [6–8]. This might be associated with their immune effect on lymphocyte activity; they work by blocking checkpoint proteins (CTLA-4, PD-1, PD-L1) and their inhibitory signal [3].

In this case report, we described the clinical management of a 67-year-old man diagnosed with locally advanced right upper lobe lung adenocarcinoma treated with anti-PD-L1 therapy (durvalumab) and after three months, a diagnosis of fibro-cavitary NTM-LD caused by *M. chimaera* in the absence of immunosuppressive treatments. We considered the onset of NTM-LD because of immunotherapy and therefore started a specific treatment for NTM, which resulted in a stable lung condition. According to the classification proposed by Morelli et al., the NTM infection described in this case report could be classified as an infection due to dysregulated immunity [3]. Indeed, it occurred after the ICI therapy started, without ongoing immunosuppressive treatment, its progression was halted by antimycobacterial treatment despite ICIs continuation and happened in a patient from a low NTM-endemicity area [6, 12].

In a systematic review of the literature, we have previously described nine cases of NTM infections by MAC or *M. abscessus* occurring during immunotherapy with ICIs and their characteristics [6]. Following this report, we strengthen the hypothesis that the administration of ICI immunotherapy may predispose to the development of progressive NTM-LD. We need prospective cohort studies to understand the actual NTM infection incidence in cancer patients undergoing immunotherapy with ICIs; unfortunately, currently available data are limited to case descriptions [6, 8]. This is particularly relevant considering the increasing incidence of NTM-LD in areas such as Western Europe or North America, where the number of patients receiving ICIs is constantly growing [12].

**Fig. 2:**
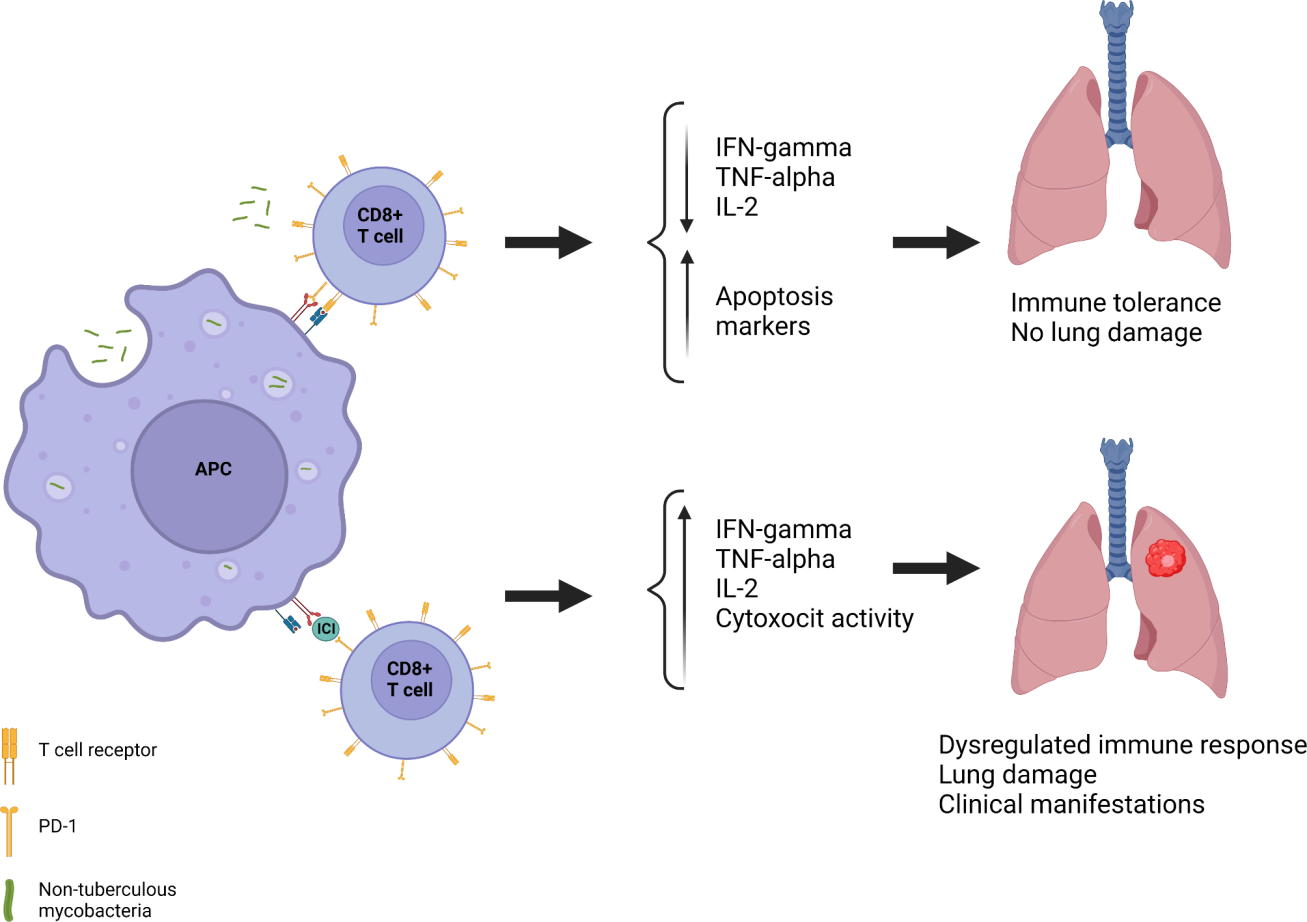
Simplified overview of the mechanism leading to NTM-LD development among patients receiving immunotherapy with immune checkpoint inhibitors. (ICI: immune checkpoint inhibitors; PD-1: programmed cell death protein 1; IFN-γ: interferon-gamma; TNF-α: tumour necrosis factor-alpha; APC: antigen-presenting cell.)

An early diagnosis to rule out NTM infection before ICI therapy in cancer patients remains challenging due to the already altered lung parenchyma and the symptoms overlapping between cancer and NTM-LD. Understanding risk factors that predispose patients to pulmonary NTM infections (such as structural and functional parenchymal abnormalities and individual exposure to environmental sources) combined with a potential increased risk when administering drugs that alter immune mechanisms should be considered by specialised physicians prescribing immunotherapy. This study aims to support those facing clinical and radiological deterioration in oncology patients receiving ICI to consider the diagnosis of NTM-LD and the possible need for antimycobacterial treatment. Figure 2 summarises the mechanisms involved in developing NTM-LD, consequently leading to a dysregulated immune response under ICI treatment.

## Conclusions

We reported the first case of NTM-LD due to *M. chimaera* in an oncologic patient receiving ICIs. We hypothesise that durvalumab treatment has induced an exaggerated immune response toward the mycobacteria, leading to immunopathology and clinical manifestations. Clinicians should be aware of this possibility in patients receiving ICIs, collectively described as immunotherapy infections due to dysregulated immunity, even without immunosuppressive treatments, particularly in countries with a high prevalence of NTM.

## Data Availability

The dataset used and/or analysed during the current study available from the corresponding author on reasonable request.
